# A Conserved Tyrosine Residue in Slitrk3 Carboxyl-Terminus Is Critical for GABAergic Synapse Development

**DOI:** 10.3389/fnmol.2019.00213

**Published:** 2019-09-10

**Authors:** Jun Li, Wenyan Han, Kunwei Wu, Yuping Derek Li, Qun Liu, Wei Lu

**Affiliations:** Synapse and Neural Circuit Research Unit, National Institute of Neurological Disorders and Stroke, National Institutes of Health, Bethesda, MD, United States

**Keywords:** Slitrk3, GABAergic synapse, gephyrin, cell adhesion molecule, GABAergic synapse development, inhibition, tyrosine, hippocampus

## Abstract

Single-passing transmembrane protein, Slitrk3 (Slit and Trk-like family member 3, ST3), is a synaptic cell adhesion molecule highly expressed at inhibitory synapses. Recent studies have shown that ST3, through its extracellular domain, selectively regulates inhibitory synapse development via the trans-synaptic interaction with presynaptic cell adhesion molecule, receptor protein tyrosine phosphatase δ (PTPδ) and the *cis*-interaction with postsynaptic cell adhesion molecule, Neuroligin 2 (NL2). However, little is known about the physiological function of ST3 intracellular, carboxyl (C)-terminal region. Here we report that in heterologous cells, ST3 C-terminus is not required for ST3 homo-dimerization and trafficking to the cell surface. In contrast, in hippocampal neurons, ST3 C-terminus, more specifically, the conserved tyrosine Y969 (in mice), is critical for GABAergic synapse development. Indeed, overexpression of ST3 Y969A mutant markedly reduced the gephyrin puncta density and GABAergic transmission in hippocampal neurons. In addition, single-cell genetic deletion of ST3 strongly impaired GABAergic transmission. Importantly, wild-type (WT) ST3, but not the ST3 Y969A mutant, could fully rescue GABAergic transmission deficits in neurons lacking endogenous ST3, confirming a critical role of Y969 in the regulation of inhibitory synapses. Taken together, our data identify a single critical residue in ST3 C-terminus that is important for GABAergic synapse development and function.

## Introduction

Synapses, the highly specialized cellular junctions, are essential for rapid chemical communication between neurons. Glutamate is the predominant excitatory neurotransmitter in the central nervous system and mainly acts on ionotropic glutamate receptors to mediate excitatory transmission. On the other hand, GABA is the dominant inhibitory neurotransmitter in the adult brain and fast inhibitory transmission is largely mediated by GABA_*A*_ receptors, a process that provides inhibitory balance to glutamatergic excitation and controls neuronal output. Accumulating evidence has shown that perturbations of synapse development and function are associated with a variety of neurological and psychiatric disorders, such as autism spectrum disorders, schizophrenia, and epilepsy (Dani et al., 2005; Scharfman, 2007; Eichler and Meier, 2008; Kehrer et al., 2008; Dudek, 2009; Gogolla et al., 2009; Markram and Markram, 2010; Rubenstein, 2010; Vattikuti and Chow, 2010; Paluszkiewicz et al., 2011; Yizhar et al., 2011; Lisman, 2012; Sheng et al., 2012). Thus, it is critical to understand the molecular mechanisms for synaptogenesis and synaptic function. While development of glutamatergic synapses has been extensively studied (Waites et al., 2005; McAllister, 2007; Kelsch et al., 2010; Clarke and Barres, 2013; Hanse et al., 2013), much less is known about the mechanisms underlying GABAergic synapse development.

Synaptic cell adhesion molecules are a class of cell surface proteins that are key players in instructing various steps of both excitatory and inhibitory synaptogenesis (Waites et al., 2005; Sudhof, 2008; Siddiqui and Craig, 2011; Lu et al., 2016; Krueger-Burg et al., 2017). Among these molecules, Slit- and Trk-like (Slitrk) proteins have been implicated in synapse development and function (Proenca et al., 2011; Won et al., 2019). Slitrks constitute a family of six members, and, among them, ST3 plays a specific role in the regulation of GABAergic synapse development, whereas other Slitrks are critical for excitatory synaptogenesis and function (Takahashi et al., 2012; Yim et al., 2013; Beaubien et al., 2016; Li et al., 2017). Molecularly, Slitrks contain two clusters of the leucine-rich repeat (LRR) domain (LRR1 and LRR2) in the amino-terminal (N-terminal) extracellular region with each cluster consisting of six LRR motif repeats, a single transmembrane domain, and a carboxyl-terminal (C-terminal) domain (Aruga and Mikoshiba, 2003). The LRR1 domain of these postsynaptic cell adhesion molecules mediates the trans-synaptic interaction with presynaptic cell adhesion molecules, receptor protein tyrosine phosphatases (PTPs), to regulate synapse development (Proenca et al., 2011; Um et al., 2014; Won et al., 2019). In addition, the LRR2 domain of ST3 has been shown to bind to another synaptic cell adhesion molecule, NL2, to regulate GABAergic synapse development (Li et al., 2017), and Slitrk1 LRR2 domain is critical for protein oligomerization (Beaubien et al., 2016). Recent studies have also identified a number of missense mutations in Slitrk N-termini that are associated with neuropsychiatric disorders (Proenca et al., 2011; Kang et al., 2016), highlighting the importance of Slitrk extracellular domains in brain development and function. However, the role of Slitrk C-termini in synapse development and transmission remains largely unclear. One prominent feature of Slitrk C-termini is that they contain several conserved tyrosine (Tyr or Y) residues (Aruga and Mikoshiba, 2003). Among them, a tyrosine residue in the distal C-termini of Slitrks, conserved between Slitrks and Trk neurotrophin receptor proteins (Y791 in human TrkA), is intriguing. In Trk receptors, neurotrophin binding leads to Tyr phosphorylation at Y791 (Reichardt, 2006), which in turn recruits phospholipase C-γ (PLC-γ) that can generate second messengers, such as IP3 and diacylglycerol (DAG), for intracellular signaling (Huang and Reichardt, 2003). However, the role of this conserved tyrosine residue in Slitrks in the regulation of synapse development and function remains unknown.

Here we have investigated the function of the C-terminus of inhibitory synaptic cell adhesion molecule, ST3, in regulating GABAergic synapses. We have found that the conserved tyrosine residue, Y969 in ST3 C-terminus, is critical for GABAergic synapse development and transmission. Mutation at this tyrosine residue impaired GABAergic synapse development and reduced inhibitory transmission, demonstrating an important role of ST3 C-terminus in the regulation of inhibitory synapses.

## Materials and Methods

### Animals

Animal housing and procedures were performed in accordance with the guidelines of the Animal Care and Use Committee (ACUC) at National Institute of Neurological Disorders and Stroke (NINDS), National Institutes of Health (NIH), and were approved by the NINDS ACUC at NIH. Adult C57BL/6 mice were purchased from Charles River, housed and bred with standard laboratory chow and water under a 12-h light/dark cycle. Mice of either sex were used in this study.

### Plasmids

Full length mouse cDNA encoding Slitrk3 (ST3) in this study was purchased from OriGene (Cat #: MR211375). Flag- or Myc-tagged full length or truncation mutants of ST3 were generated by overlapping PCR and were subcloned into pcDNA3.0 expression vector, respectively. Y969A point mutation (TAC→GCA) in ST3 was generated by overlapping PCR and subcloned into pcDNA3.0 expression vector. To screen the ST3 single-guidance RNA (sgRNA) sequences for single-cell knockout experiment, we have designed 3 sgRNA sequence candidates using online tools^1^. The primer sequences are as shown below:

ST3 #1: forward, 5′-CACCgAGCTGTTTCCTTAACGCA TC-3′;
reverse, 5′-AAACGATGCGTTAAGGAAACAGCTc-3;
ST3 #2: forward 5′-CACCgACGAAGGTCCAGATGCGT TA-3′;
reverse 5′-AAACTAACGCATCTGGACCTTCGTc-3′;
ST3 #3: forward 5-CACCgCAATAGTGCGCACATCAC GG-3;
reverse 5-AAACCCGTGATGTGCGCACTATTGc-3.

The human codon-optimized Cas9 and chimeric sgRNA expression plasmid (pSpCas9 BB-2A-GFP, or pX458) was purchased from Addgene (#48138, Ran et al., 2013). To generate sgRNA plasmids, a pair of annealed oligos were ligated into the sgRNA scaffold of pX458. To examine the specificity of the single-cell knockout effect on GABAergic synapses, sgRNA resistant ST3 plasmids were constructed for rescue experiments. The constructs of Flag- or Myc-tagged ST3, which were resistant to ST3 sgRNA#2, were generated by overlapping PCR to make five-point mutations in the ST3 sgRNA#2-targeting site (mutation region: ACGAAGGTCCAGATGCGTTA to ACGATGGACCTGAAGCATTA; amino acids: Asn-Ala-Ser-Gly-Pro-Ser) and then subcloned into the pcDNA3.0 plasmid, respectively. All constructs were verified by DNA sequencing.

### Cell Culture and Transfection

HEK293T and COS7 cells were grown in DMEM (GIBCO) supplemented with 10% fetal bovine serum (FBS) (GIBCO), 1% Pen/Strep, 1% Glutamine, and 1% sodium pyruvate, in a humidified atmosphere in a 37°C incubator with 5% CO_2_. Transfection was performed in 6 cm dishes with indicated cDNAs using CalPhos Mammalian Transfection Kit (Clontech, 631312) or Lipofectamine 3000 Transfection Reagent (Invitrogen, L3000015), following the manufacturer’s instructions.

### Dissociated Hippocampal Neuronal Culture

Hippocampal neuronal cultures were prepared from E18 time-pregnant C57BL/6 mice as previously described (Gu et al., 2016). Briefly, the embryonic mouse hippocampi were dissected out in ice-cold Hank’s balanced salt solution, and digested in papain (Worthington, LK003176) solution at 37°C for 45 min. After centrifugation for 5 min at 800 rpm, the pellet was resuspended in DNase I-containing Hank’s solution, and then mechanically dissociated into single cells by gentle trituration using a pipette. Digestion was stopped by adding trypsin inhibitor (10 mg/ml, Sigma T9253) and BSA (10 mg/ml, Sigma A9647), and then centrifuged at 800 rpm for 10 min. The pellet was resuspended in Neurobasal media containing 2% B27 supplements and L-glutamine (2 mM). Dissociated neurons were plated at a density of 1.5∼2.0 × 10^5^ cells/well on poly-D-lysine (Sigma P7886)-coated 12 mm glass coverslips residing in 24-well plates for electrophysiology recording, and a lower plating density (1.0∼1.5 × 10^5^ cells/well) was adopted when neurons were used for immunocytochemistry. Culture media were changed by a half volume once a week.

### Neuronal Transfection

For sgRNA transfection, hippocampal neurons were transfected at day 2–3 *in vitro* (DIV2-3) using a modified calcium phosphate transfection as described previously (Li et al., 2019). Briefly, 5 μg total cDNA was used to generate 200 μL total precipitates, which was added to each well at a 40 μL volume (five coverslips/group). After 2-h incubation in a 37°C incubator, the transfected cells were incubated with pre-warmed, 10% CO_2_ pre-equilibrated Neurobasal medium, and placed in a 37°C, 5% CO_2_ incubator for 20 min to dissolve the calcium-phosphate particles. The coverslips were then transferred back to the original conditioned medium. The cells were cultured to DIV 14-16 before experiments. For overexpression experiments for both staining and electrophysiological recordings, neuronal transfection was performed at DIV12-13 in 24-well plate with indicated cDNAs using Lipofectamine 3000 Reagent following the manufacturer’s instructions. Neurons after transfection were analyzed at DIV 14-16.

### Immunocytochemistry

The cells grown on coverslips were rinsed with PBS twice and fixed in 4% paraformaldehyde (PFA)/4% sucrose/1× PBS solution for 15 min at RT, followed by permeabilization with 0.2% TritonX-100/1× PBS for 15 min. Subsequently, cells were blocked with 5% normal goat serum in 1× PBS for 1 h. Cells were incubated with primary antibodies as follows: anti-Myc (1:1,000, ab18185, Abcam), anti-Flag M2 (1:1,000, F3165, Sigma), anti-Flag (1:1,000, F7425, Sigma), anti-Gephyrin (1:500, 147018, Synaptic Systems), anti-Gephyrin (1:500, 147021, Synaptic Systems), anti-Slitrk3 (1:1,000, ABN356, Sigma) and anti-MAP2 (1:1,000, MAP, Aves Labs) in 1× PBS solutions overnight at 4°C. Cells were washed three times with 1× PBS and then incubated with Alexa Fluor 488, 555, or 647-conjugated IgG for 30 min. Coverslips were washed for three times with 1× PBS and mounted with Fluoromount-G (Southern Biotech) for imaging acquisition.

### Co-immunoprecipitation and Western Blot

For co-immunoprecipitation (Co-IP) experiments, the indicated constructs were transfected into HEK293T cells by calcium phosphate transfection. After transfection for 48 h, cells were homogenized in ice-cold lysis buffer containing 25 mM Tris (pH 7.4), 1% Triton X-100, 150 mM NaCl, 5% glycerol, 1 mM EDTA, and EDTA-free protease inhibitors (Roche, 5892791001). Equal amounts of cell lysates were incubated with anti-Flag M2 affinity gel (Sigma, A2220) overnight at 4°C. Beads were washed three times with 500 μl lysis buffer and diluted in an equal amount of 2 × loading buffer (Bio-Rad 161-0737) containing 10% β-mercaptoethanol (Fisher Scientific BP176100) and denatured for 5 min at 95°C. Proteins were separated on 10% SDS-PAGE (Bio-Rad), and transferred onto PVDF membrane for immunoblotting with indicated antibodies. For the dimerization experiment, Flag-ST3 and Myc-ST3 or Myc-ST3 ΔCT plasmids were co-transfected into HEK293T cells for 48 h. Anti-Myc (1:1,000, 2278, Cell Signaling Technology) and anti-Flag (1:1,000, F2555, Sigma) antibodies were used in this experiment. For ST3 sgRNA screening and resistant plasmid verification experiments, Myc-ST3 or resistant mutants and sgRNA candidates were co-transfected in a ratio at 1:2 to HEK293T cells (2 × 10^6^ cells/well on transfection day in 6-well plate), while empty pcDNA3.0 vector was added to balance the total amount of DNA in single transfection conditions. Proteins were detected with anti-Slitrk3 antibody (1:1,000, ABN356, Sigma) or anti-α-tubulin antibody (1:5,000, T8203, Sigma) by enhanced chemiluminescence (ECL) method.

### Image Acquisition and Analysis

Fluorescence images were acquired with a Zeiss LSM 880 laser scanning confocal microscope using a 63× oil-immersion objective lens (numerical aperture 1.4). For fluorescent intensity analysis in both of COS7 cells and neurons, the maximal intensity projected images were generated by ZEN software (Zeiss) from seven serial optical sections, and the mean fluorescent intensity of region of interest (ROI) was measured following the subtraction for off-cell background with ImageJ software. For gephyrin and vGAT puncta density analysis, confocal images from 1 to 3 secondary or tertiary dendrites (35 μm in length) per neuron from at least ten neurons in each group were collected and quantified by counting the number of puncta per 10 μm dendrites with ImageJ puncta analyzer program. Thresholds were set at 3 SDs above the mean staining intensity of six nearby regions in the same visual field. Thresholded images present a fixed intensity for all pixels above threshold after having removed all of those below. Labeled puncta were defined as areas containing at least four contiguous pixels after thresholding. For co-localization analysis, the gephyrin-positive Myc clusters indicate the number of Myc clusters exhibiting at least partial pixel overlapping with thresholded gephyrin clusters, and co-localization percentage was quantified by the measurement of gephyrin-positive Myc clusters compared to the total number of thresholded Myc clusters.

### Electrophysiology

For mIPSC recording in dissociated hippocampal neuronal cultures, neurons grown on coverslips were transferred to a submersion chamber on an upright Olympus microscope, and perfused with ACSF solution supplemented with TTX (0.5 μM), DNQX (20 μM), and strychnine (1 μM). GFP fluorescent positive neurons in neuronal cultures were identified by epifluorescence microscopy. Neurons were voltage-clamped at −70 mV for detection of mIPSC events. The intracellular solution for GABAergic mIPSC recording contained (in mM) CsMeSO_4_ 70, CsCl 70, NaCl 8, EGTA 0.3, HEPES 20, MgATP 4, and Na_3_GTP 0.3. Osmolality was adjusted to 285–290 mOsm and pH was buffered at 7.25–7.35. Series resistance was monitored and not compensated, and cells in which series resistance varied by 25% during a recording session were discarded. Synaptic responses were collected with a Multiclamp 700B amplifier (Axon Instruments, Foster City, CA, United States), filtered at 2 kHz, and digitized at 10 kHz. All recordings were performed at RT. 100–300 consecutive miniature events were semi automatically detected by off-line analysis using customized software Igor Pro (Wavemetrics) as described before (Milstein et al., 2007; Lu et al., 2009, 2013; Herring et al., 2013), using a threshold of 6 pA. All mIPSC events were visually inspected to ensure that they were mIPSCs during analysis, and non-mIPSC traces were discarded. All pharmacological reagents were purchased from Abcam, and other chemicals were purchased from Sigma.

### Statistical Analysis

Statistical analysis was performed in GraphPad Prism 7.0. Direct comparisons between two groups were made using two-tailed, unpaired Student’s *t*-test. Multiple group comparisons were made using one-way analysis of variance (ANOVA) with *post hoc* Fisher’s LSD test. The significance of cumulative probability distributions was assessed by Kolmogorov–Smirnov (K–S) test. The difference was considered significant at levels of *p* < 0.05 (^∗^), *p* < 0.01 (^∗∗^), *p* < 0.001 (^∗∗∗^), or *p* < 0.0001 (^∗∗∗∗^), respectively. *p*-values ≥ 0.05 were considered not significant. All data *n* in the text and figures were presented as Mean ± SEM (standard error of mean).

## Results

**FIGURE 1:**
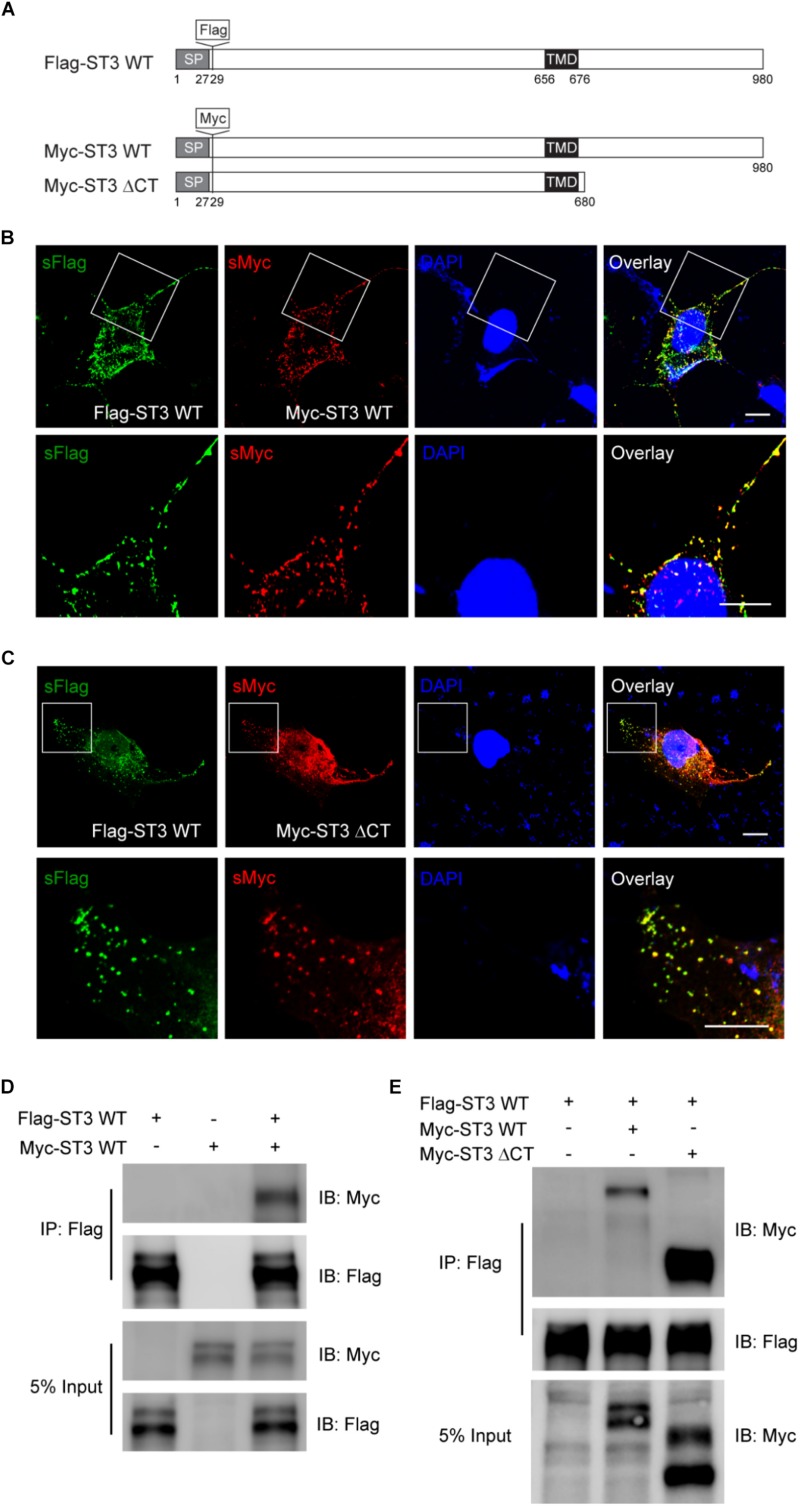
ST3 C-terminus is not required for ST3 homo-dimerization in heterologous cells. **(A)** Schematic of Flag-ST3 WT, Myc-ST3 WT, and Myc-ST3 ΔCT. Flag or Myc tag was inserted at amino acid 29. Signal peptide (SP). **(B,C)** Representative images (top panel, low magnification; bottom panel, high magnification of the boxed area at the top) showing surface (s) Flag-ST3 co-localized with surface Myc-ST3 **(B)** or surface Myc-ST3 ΔCT **(C)** in COS7 cells. Scale bar, 10 μm. **(D,E)** Co-IP assay of Flag-ST3 with Myc-ST3 **(D)** or Myc-ST3 ΔCT **(E)** in HEK293T cells. Cell lysates from HEK293T cells transfected with Flag-ST3, Myc-ST3, or Flag-ST3 together with Myc-ST3 or Myc-ST3 ΔCT, were immunoprecipitated with agarose beads conjugated with anti-Flag antibody, and then probed with indicated antibodies. IB, immunoblotting. Both Myc-ST3 **(D)** or Myc-ST3 ΔCT **(E)** were co-IPed with Flag-ST3. *N* = 3 independent repeats.

We first examined whether ST3 could form homo-dimers in heterologous cells and whether ST3 C-terminus was important in this process, as dimerization is a common feature for transmembrane protein-mediated signaling (Maruyama, 2015) and is important for cell adhesion molecules in promoting synapse development and function (Ko et al., 2009; Fogel et al., 2011; Shipman and Nicoll, 2012). Toward this end, we generated Flag or Myc tagged ST3 WT at its N-terminus (Flag-ST3 WT, Myc-ST3 WT) and Myc tagged ST3 C-terminal deletion mutant (Myc-ST3 ΔCT), as shown in Figure 1A. We then performed immunocytochemical experiments in COS7 cells expressing both Flag-ST3 WT and Myc-ST3 WT and examined the distribution of surface ST3. We found that surface Flag-ST3 WT co-localized with Myc-ST3 WT at distinct puncta (Figure 1B), indicating that these two molecules localize at the same subcellular compartments. Interestingly, ST3 Myc-ST3 ΔCT lacking the majority of C-terminus also co-localized with Flag-ST3 WT at the cell surface (Figure 1C), indicating that ST3 C-terminus is not critical for the co-localization of tagged ST3 in heterologous cells. We also conducted co-immunoprecipitation assays in HEK293T cells expressing both Flag-ST3 WT and Myc-ST3 WT or expressing either plasmids on its own. We found that a Flag antibody could pull down Myc-ST3 WT from cells expressing both constructs, but not from control cells expressing either one (Figure 1D), showing that ST3 can form homo-dimers in heterologous cells. To probe whether ST3 C-terminus was important for homo-dimerization, we co-transfected Myc-ST3 ΔCT together with Flag-ST3 WT in HEK293T cells. Co-immunoprecipitation assays showed that both Myc-ST3 and Myc-ST3 ΔCT were co-immunoprecipitated with Flag-ST3 (Figure 1E), indicating that ST3 C-terminus is not required for ST3 homo-dimerization. Together, these data show that ST3 forms dimers in a C-terminus independent manner in heterologous cells.

**FIGURE 2:**
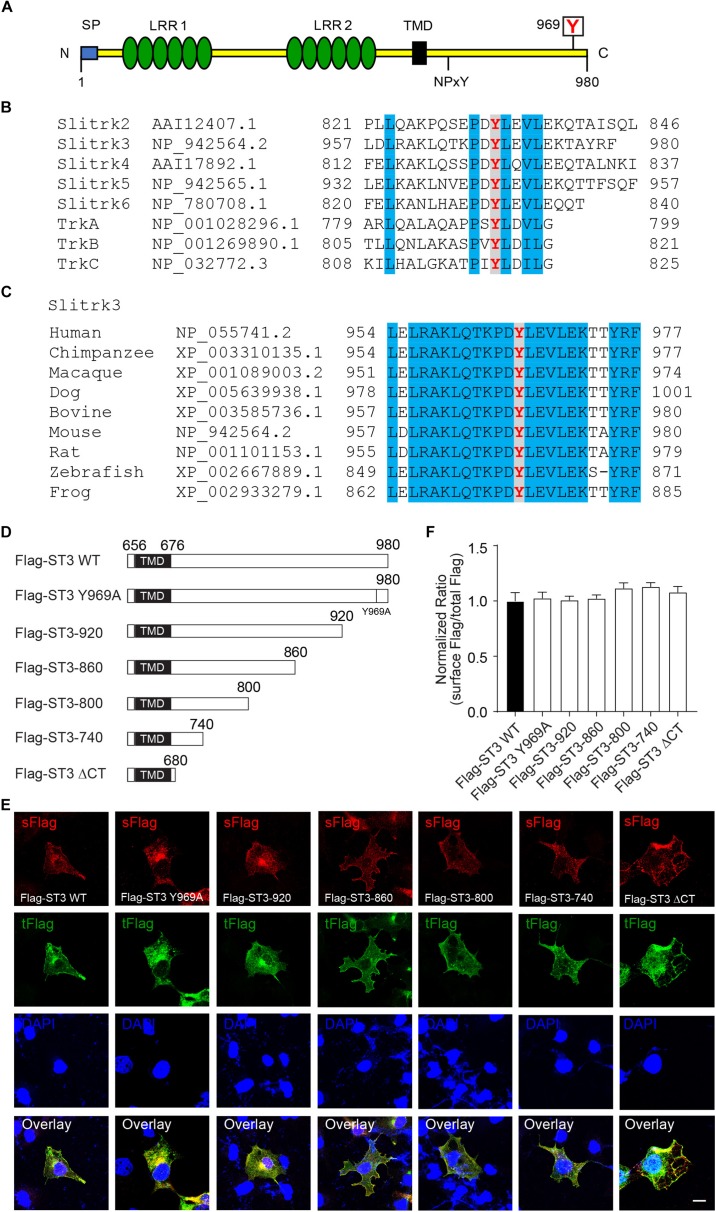
ST3 C-terminus is not necessary for surface expression of ST3 in heterologous cells. **(A)** Schematic of ST3 showing LRR1 and LRR2 clusters in its extracellular region, and the intracellular conserved tyrosine residues. N, N-terminus; C, C-terminus; SP, signal peptide; LRR1, leucine-rich repeats cluster 1; LRR2, leucine-rich repeats cluster 2; TMD, transmembrane domain; NPxY, NPxY motif; boxed Y, a conserved tyrosine residue in Slitrks and Trk receptors. **(B)** Amino acid sequence alignment of the C-termini of mouse Slitrk and Trk proteins. The tyrosine in red indicates the conserved residues (Y969 in mouse ST3) in Slitrks and Trk receptors, and residues in blue indicate other conserved amino acid residues between Slitrks and Trk receptors. **(C)** Cross species alignment of the ST3 C-termini. The tyrosine in red indicates the conserved residues (Y969 in mouse ST3) in ST3 C-termini from nine different vertebrate species, and residues in blue indicate other conserved amino acid residues across different species in the distal Slitrk3 C-termini. **(D)** Schematic of WT and C-terminal mutant forms of Flag-ST3. TMD, transmembrane domain. **(E,F)** Representative images showing surface (*s*) and total (*t*) Flag expressions of Flag-ST3 WT or Flag-ST3 mutants in COS7 cells. The ratios of surface to total fluorescent intensity were calculated and showed that ST3 C-terminus was not required for ST3 expression at the cell surface (*n* ≥ 14 for each group, One-way ANOVA test, *p* > 0.05, *N* = 3 independent experiments). Scale bar, 10 μm.

To determine whether ST3 C-terminus was important for trafficking of ST3 to the cell surface in heterologous cells, we generated a series of mutants of Flag-ST3 in its C-terminus (Figure 2D) and expressed them individually in COS7 cells. We then performed immunocytochemical experiments to examine the surface expression of WT and truncated Flag-ST3 mutants by measuring the ratio of surface Flag fluorescence to total Flag fluorescence. As shown in the Figures 2E,F, all Flag-ST3 truncation mutants showed similar expression levels on the cell surface as compared to WT Flag-ST3. In fact, the surface expression levels of Flag-ST3ΔCT, lacking the majority of C-terminus (truncation at 680), was comparable to WT Flag-ST3 (Figures 2E,F). We noticed that in the C-terminus of ST3, there is an evolutionarily conserved tyrosine residue at the position of 969 (Figures 2A–C), which shows homology with Trk receptors. We mutated the conserved tyrosine residue in Y969 to alanine (Flag-ST3 Y969A) and found that expression of this mutant on the cell surface was similar to WT Flag-ST3 (Figures 2E,F). Taken together, Figures 1, 2 show that, while ST3 can form dimers and traffic to the cell surface in heterologous cells, its C-terminus is not critical in these processes.

**FIGURE 3:**
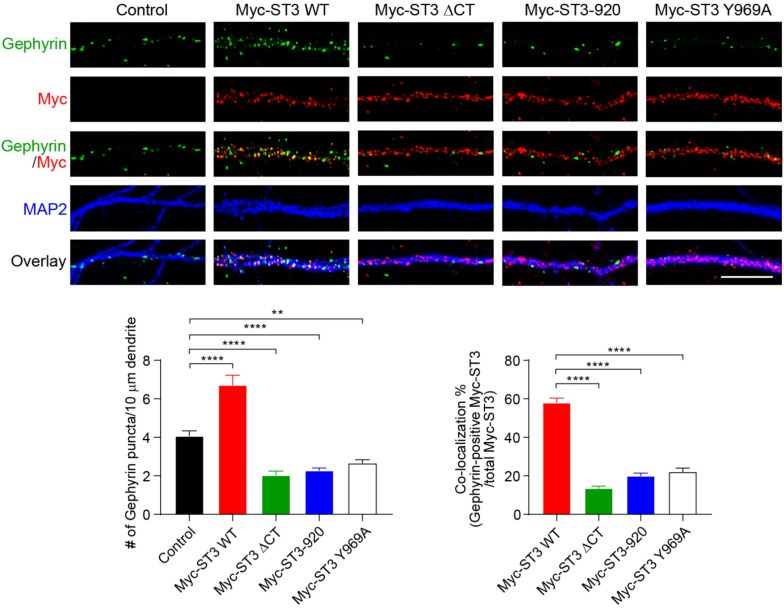
Identification of a critical residue Y969 in ST3 C-terminus that is important for GABAergic synapse development in hippocampal neurons. Representative images of dendrites (top) and quantification analysis (bottom) showed that overexpression of WT Myc-ST3 significantly increased gephyrin puncta density in cultured hippocampal neurons, whereas overexpression of Myc-ST3 ΔCT, Myc-ST3-920, in which the last 60 amino acids of ST3 were deleted, or the Myc-ST3 Y969A mutant significantly decreased gephyrin puncta density (Control, 4.07 ± 0.27, *n* = 12; Myc-ST3 WT, 6.72 ± 0.52, *n* = 12; Myc-ST3 ΔCT, 2.03 ± 0.22, *n* = 9; Myc-ST3-920, 2.28 ± 0.13, *n* = 18; Myc-ST3 Y969A, 2.66 ± 0.18, *n* = 11. One-way ANOVA test, ^∗∗∗∗^*p* < 0.0001, ^∗∗^*p* < 0.01. *N* = 3 independent experiments). Overexpression of Myc-ST3 Y969A also significantly decreased co-localization between ST3 and gephyrin (percentage of co-localization: Myc-ST3 WT, 57.96 ± 2.38, *n* = 12; Myc-ST3 ΔCT, 13.59 ± 1.03, *n* = 9; Myc-ST3-920, 19.98 ± 1.45, *n* = 18; Myc-ST3 Y969A, 22.04 ± 1.98, *n* = 11. One-way ANOVA test, ^∗∗∗∗^*p* < 0.0001. *N* = 3 independent experiments). Scale bar, 10 μm.

To further examine the role of ST3 C-terminus, we investigated the function of ST3 C-terminus in the regulation of GABAergic synapses in hippocampal neuronal cultures, as ST3 is a key inhibitory synaptic cell adhesion molecule (Takahashi et al., 2012; Yim et al., 2013; Li et al., 2017). We first overexpressed WT Myc-ST3 and Myc-ST3 ΔCT in dissociated hippocampal cultures, and examined the density of gephyrin, an inhibitory postsynaptic marker and scaffold protein (Tretter et al., 2012; Tyagarajan and Fritschy, 2014), in neuronal dendrites. We found that, in neurons overexpressing WT Myc-ST3, the gephyrin density was significantly increased (Figure 3), which is in agreement with a previous study (Yim et al., 2013). However, in neurons overexpressing Myc-ST3 ΔCT that lacked the majority of C-terminus, the density of gephyrin puncta was significantly reduced, as compared to control neurons (Figure 3), suggesting that ST3 C-terminus is critical for the regulation of GABAergic synapse density by ST3. Interestingly, overexpression of a ST3 mutant, Myc-ST3-920, in which the last 60 amino acids after the residue 920 (including the conserved Y969) were deleted, also strongly decreased the gephyrin density (Figures 2D, 3). This indicates that the sequence after amino acid 920 harbors the functional domain important for ST3 to regulate neuronal gephyrin density. Furthermore, to determine whether the Y969 residue in the distal C-terminus was critical (Figures 2A–C), we expressed the Myc-ST3 Y969A mutant in hippocampal cultures. We found that the gephyrin density was significantly reduced in neurons expressing this mutant (Figure 3), similar to Myc-ST3 ΔCT and Myc-ST3-920. In addition, compared to Myc-ST3 WT, co-localization of Myc-ST3 ΔCT, Myc-ST3-920, or Myc-ST3 Y969A with gephyrin was significantly impaired (Figure 3).

**FIGURE 4:**
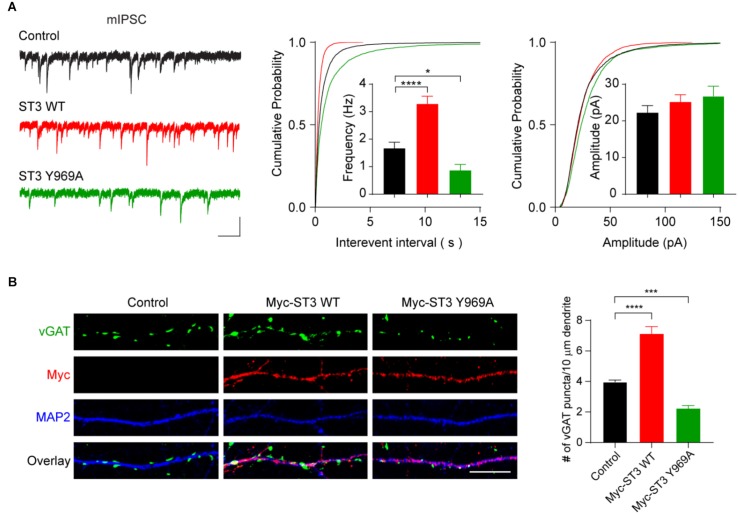
Y969 in ST3 C-terminus is critical for GABAergic synaptic transmission. **(A)** mIPSC recording showed that overexpression of WT Myc-ST3 (co-expressed with GFP) significantly increased mIPSC frequency, whereas overexpression of the Myc-ST3 Y969A mutant significantly reduced the frequency of mIPSCs in hippocampal cultured neurons. Insets showed the mean ± SEM of mIPSC frequency and amplitude, respectively [Frequency (Hz): Control, 1.67 ± 0.22, *n* = 15; ST3 WT, 3.29 ± 0.28, *n* = 13; ST3 Y969A, 0.87 ± 0.21, *n* = 12. One-way ANOVA test, ^∗∗∗∗^*p* < 0.0001, ^∗^*p* < 0.05. Kolmogorov–Smirnov (K–S) test, *p* < 0.0001 between Control and ST3 WT or ST3 Y969A for interevent interval. Amplitude (pA): Control, 22.29 ± 1.87, *n* = 15; ST3 WT, 25.18 ± 1.96, *n* = 13; ST3 Y969A, 26.78 ± 2.67, *n* = 12. One-way ANOVA test, *p* > 0.05. K–S test, *p* < 0.05 between Control and ST3 WT for amplitude, *p* < 0.0001 between Control and ST3 Y969A for amplitude. *N* = 3 independent experiments]. Scale bar, 20 pA and 1 s. **(B)** Representative images of dendrites (left) and quantification analysis (right) showed that overexpression of Myc-ST3 WT significantly increased vGAT puncta density in cultured hippocampal neurons, whereas overexpression of Myc-ST3 Y969A mutant significantly decreased vGAT puncta density (Control, 3.97 ± 0.13, *n* = 14; Myc-ST3 WT, 7.13 ± 0.46, *n* = 11; Myc-ST3 Y969A, 2.23 ± 0.19, *n* = 12. One-way ANOVA test, ^∗∗∗∗^*p* < 0.0001, ^∗∗∗^*p* < 0.001. *N* = 3 independent experiments), Scale bar, 10 μm.

The reduction of gephyrin puncta in neurons expressing ST3 mutants, as shown in Figure 3, suggested impairment of GABAergic transmission in these neurons. To examine this, we performed whole-cell recordings to measure miniature inhibitory postsynaptic currents (mIPSCs) in hippocampal neurons overexpressing WT ST3 or the ST3 Y969A mutant in C-terminus (also simultaneously expressing GFP). In neurons overexpressing WT ST3, the frequency, but not amplitude, of mIPSCs was significantly increased (Figure 4A). In contrast, in neurons overexpressing ST3 Y969A, GABAergic transmission was strongly reduced (Figure 4A). Specifically, mIPSC frequency was decreased by ∼50% (Figure 4A), indicating a key role of ST3 Y969 in the regulation of inhibitory transmission. In addition, we found that vGAT density was reduced in neurons overexpressing Myc-ST3 Y969A (Figure 4B), indicating a reduction of GABAergic synapse density, consistent with the decrease of mIPSC frequency in these cells (Figure 4A).

**FIGURE 5:**
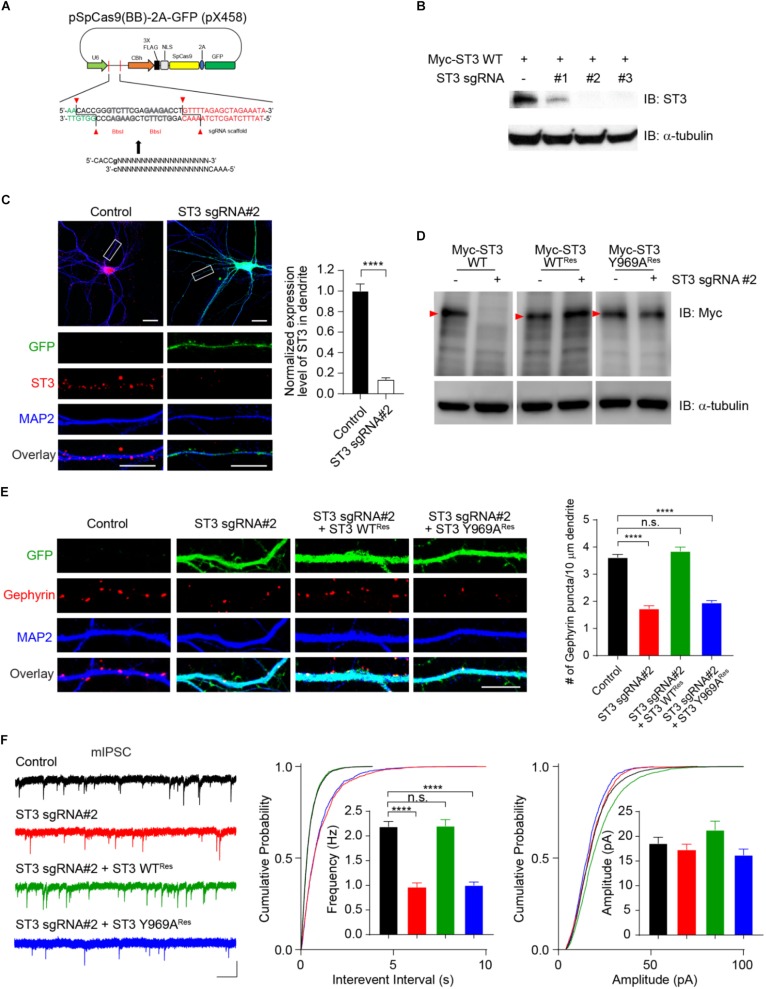
Single-cell genetic deletion and rescue of ST3 reveal the importance of Y969 in the regulation of GABAergic transmission. **(A)** Schematic diagram of CRISPR/Cas9 vector (simultaneously expresses GFP) targeting *ST3* gene loci in mouse genome. **(B)** Screening of knockout effect of candidate sgRNAs in HEK293T cells. Western blot analysis showed that sgRNA#2 and sgRNA#3, but not sgRNA#1, strongly reduced ST3 expression in HEK293T cells. α-tubulin was used as an internal control. *N* = 3 independent repeats. **(C)** Confocal images and quantification analysis showed a significant decrease of ST3 expression in the dendritic region of hippocampal neurons expressing sgRNA#2 (Control, 1.0 ± 0.07, *n* = 12; ST3 sgRNA#2, 0.14 ± 0.02, *n* = 12, unpaired *t*-test, ^∗∗∗∗^*p* < 0.0001. *N* = 3 independent repeats). Scale bar, 20 mm (top) and 10 mm (bottom). **(D)** Western blot analysis validated the expression of sgRNA#2 resistant WT ST3 (ST3 WT^*Res*^) and the Y969A mutant (ST3 Y969A^*Res*^) in HEK293T cells. α-tubulin was used as an internal control. Red arrow heads indicated Myc-ST3 protein bands. *N* = 3 independent repeats. **(E)** Representative images and quantification analysis showed that the decrease of gephyrin puncta density in hippocampal neurons expressing ST3 sgRNA#2 could be rescued by co-expressing ST3 WT^*Res*^, but not ST3 Y969A^*Res*^ (Control, 3.62 ± 0.11, *n* = 15; ST3 sgRNA#2, 1.73 ± 0.10, *n* = 13; ST3 sgRNA#2 + ST3 WT^*Res*^, 3.85 ± 0.14, *n* = 13; ST3 sgRNA#2 + ST3 Y969A^*Res*^, 1.96 ± 0.07, *n* = 14. One-way ANOVA test, ^∗∗∗∗^*p* < 0.0001. *N* = 3 independent repeats). Scale bar, 10 mm. **(F)** mIPSC recording data showed that sgRNA#2 resistant WT ST3, but not ST3 Y969A, could fully rescue GABAergic transmission deficits in hippocampal cultured neurons expressing sgRNA#2. Insets displayed the mean ± SEM frequency and amplitude, respectively [Frequency (Hz): Control, 2.19 ± 0.11, *n* = 12; ST3 sgRNA#2, 0.96 ± 0.09, *n* = 10; ST3 sgRNA#2 + ST3 WT^*Res*^, 2.2 ± 0.13, *n* = 10; ST3 sgRNA#2 + ST3 Y969A^*Res*^, 1.0 ± 0.06, *n* = 10. One-way ANOVA test, ^∗∗∗∗^*p* < 0.0001. K–S test, *p* < 0.0001 between Control and ST3 sgRNA#2 or ST3 sgRNA#2 + ST3 Y969A^*Res*^ for interevent interval. Amplitude (pA): Control, 18.59 ± 1.23, *n* = 12; ST3 sgRNA#2, 17.25 ± 1.18, *n* = 10; ST3 sgRNA#2 + ST3 WT^*Res*^, 21.31 ± 1.79, *n* = 10; ST3 sgRNA#2 + ST3 Y969A^*Res*^, 16.21 ± 1.21, *n* = 10. One-way ANOVA test, *p* > 0.05. K–S test, *p* < 0.0001 between Control and ST3 sgRNA#2 + ST3 WT^*Res*^ for amplitude, *p* < 0.001 between Control and ST3 sgRNA#2 + ST3 Y969A^*Res*^ for amplitude. *N* = 3 independent repeats]. Scale bar, 20 pA and 1 s.

To further characterize the role of ST3 Y969 in the regulation of GABAergic synapses, we performed single-cell knockout (KO) and rescue experiments. To this end, we employed the CRISPR-Cas9 system to develop three single-guide RNAs (sgRNAs) to target *ST3* gene loci in the mouse genome (Figure 5A). In HEK293T cells, Western blot experiments showed that, among the three candidates, sgRNA candidate 1 (sgRNA#1) only partially reduced the expression of co-transfected Myc-ST3, while both sgRNA#2 and sgRNA#3 strongly decreased the expression levels of Myc-ST3 (Figure 5B). We further probed the effectiveness of sgRNA#2 in hippocampal neuronal cultures by performing immunocytochemical assays. We found that expression of sgRNA#2 (the vector also simultaneously expresses GFP) strongly diminished endogenous ST3 in neurons, as compared to control cells, showing that sgRNA#2 was an effective candidate in targeting endogenous ST3 (Figure 5C). To study the specificity of the effect of sgRNA#2-mediated KO on GABAergic synapses, we also developed sgRNA#2-resistant ST3 mutants (ST3 WT^*Res*^ and ST3 Y969A^*Res*^) (Figure 5D).

We found that, in hippocampal cultured neurons expressing ST3 sgRNA#2, gephyrin puncta density was significantly reduced (Figure 5E), consistent with previous studies using ST3 knockdown approaches (Takahashi et al., 2012; Yim et al., 2013; Li et al., 2017). Importantly, co-expression of sgRNA#2 with ST3 WT^*Res*^ fully restored the gephyrin puncta deficits (Figure 5E), showing that the effect of sgRNA#2 on gephyrin puncta is due to the loss of ST3 protein. Strikingly, co-expression of sgRNA#2 with ST3 Y969A^*Res*^ could not rescue the deficits of gephyrin puncta density (Figure 5E), demonstrating the critical importance of Y969 in determining the function of ST3 in the regulation of gephyrin puncta density in hippocampal neurons.

Electrophysiological measurement of mIPSCs in hippocampal cultured neurons expressing sgRNA#2 further demonstrated that there was a strong reduction of mIPSC frequency, but not amplitude (Figure 5F), in agreement with previous studies using ST3 shRNA knockdown or germline ST3 KO approaches (Takahashi et al., 2012; Yim et al., 2013; Li et al., 2017). To examine the specificity of single-cell ST3 KO on GABAergic transmission, we performed rescue experiments by co-expressing sgRNA#2 and ST3 WT^*Res*^ and measured mIPSCs. We found that ST3 WT^*Res*^ fully rescued deficits of mIPSC frequency (Figure 5F). To determine the role of ST3 Y969 in the regulation of GABAergic transmission, we co-expressed sgRNA#2 and ST3 Y969A^*Res*^ and examined mIPSCs. We found that ST3 Y969A^*Res*^ could not rescue inhibitory transmission in neurons expressing sgRNA#2 (Figure 5F), consistent with the cell biological data of gephyrin puncta density (Figure 5E). Taken together, these data show that Y969A mutation abolishes ST3 function in regulating GABAergic synapses and reveal a novel mechanism for ST3-mediated synapse development.

## Discussion

Previous studies have demonstrated that, among the six Slitrk family members Slitrk1-6, ST3 is an inhibitory postsynaptic adhesion molecule critical for GABAergic synapse development and function (Takahashi et al., 2012; Yim et al., 2013). Recently, we have further shown that ST3 plays a temporal specific role in the regulation of GABAergic synaptogenesis in hippocampal neurons (Li et al., 2017). Indeed, ST3 is important for GABAergic synapse development in more mature, but not in developing, hippocampal neurons in culture (Li et al., 2017). Importantly, the regulation of GABAergic synapse development by ST3 requires ST3 C-terminus (Li et al., 2017), highlighting the importance of ST3 C-terminus-mediated signaling in development of GABAergic connections. However, the molecular analysis and functional dissection of ST3 C-terminus in ST3 function and synapse development have not been investigated.

Our data demonstrate that, while ST3 C-terminus is not critical for a variety of ST3 functions in heterologous cells, it is important for GABAergic synapse development in hippocampal neurons. Indeed, we found that ST3 forms homodimers in a C-terminus independent manner in heterologous cells, suggesting that other domains of ST3 are critical for its dimerization. This is consistent with a recent report that the second LRR cluster in the extracellular region of Slitrk1 is necessary for Slitrk1 homo-dimerization (Beaubien et al., 2016). In addition, in heterologous cells, ST3 C-terminus is dispensable for its trafficking to the cell surface. In contrast, through both overexpression and molecular replacement approaches in hippocampal neurons, we have identified a single, evolutionarily conserved amino acid, Y969, in the ST3 C-terminus, which is crucial for ST3 function in the regulation of inhibitory synapse development. Specifically, overexpression of WT ST3 increases gephyrin or vGAT puncta and enhances inhibitory transmission, but overexpression of ST3 Y969A mutant in hippocampal neuronal cultures strongly reduces the density of gephyrin or vGAT puncta and significantly decreases GABAergic transmission. This suggests that Y969A acts as a dominant negative mutant of ST3. Furthermore, while co-expression of WT ST3 could rescue gephyrin puncta density and GABAergic transmission deficits in ST3 KO neurons, the Y969A mutant could not, showing that the mutation at Y969 inactivates ST3 function in promoting GABAergic synapse development. Thus, Y969-mediated signaling is critical for the regulation of GABAergic synapse development by ST3.

Currently, the molecular mechanisms underlying ST3 Y969-mediated signaling for inhibitory synapse development remain unclear. Y969 is a conserved tyrosine residue in Slitrk2-6 and Trk neurotrophin receptor proteins (Figures 2A–C; Aruga and Mikoshiba, 2003). In TrkA receptors, ligand binding leads to Tyr phosphorylation at Y791, the homologous conserved tyrosine in human TrkA (Reichardt, 2006). Functionally, Y791 phosphorylation in TrkA can recruit the signaling molecule, PLC-γ, which in turn generates second messengers such as IP3 and DAG for intracellular signaling (Reichardt, 2006). Similarly, analogous sites in TrkB and TrkC also undergo phosphorylation and initiate PLC-γ-mediated signaling (Reichardt, 2006). It remains unknown as to whether ST3 Y969 can be phosphorylated and whether PLC-γ-mediated signaling is involved in ST3 function in neurons. Interestingly, a recent study has shown that Slitrk5 can interact with TrkB in a BDNF-dependent manner (Song et al., 2015), raising the possibility that Slitrks, including ST3, might be substrates of Trk tyrosine kinases.

Recent structure and function analysis have identified several functional domains in ST3 that are important for ST3 in the regulation of inhibitory synapse development. For instance, ST3 extracellular LRR domains, likely the LRR1 cluster, mediate the interaction with presynaptic cell adhesion molecule, PTPδ, for induction of inhibitory synapse differentiation (Takahashi et al., 2012; Yim et al., 2013; Um et al., 2014). The LRR9 in the LRR2 cluster of ST3 binds to NL2 and the ST3-NL2 interaction is critical for development of GABAergic innervations at the late developmental stages (Li et al., 2017). We have now shown that a single conserved residue, Y969 in the ST3 C-terminus, is critical for the function of ST3 in the regulation of inhibitory synapse development. Understanding the regulatory mechanisms underlying the ST3 Y969-mediated signaling in the future will help reveal the molecular pathways for constructing inhibitory neural circuits in the brain.

## Data Availability

All datasets generated for this study are included in the manuscript and/or the supplementary files.

## Ethics Statement

Animal housing and procedures were performed in accordance with the guidelines of the Animal Care and Use Committee (ACUC) at National Institute of Neurological Disorders and Stroke (NINDS), National Institutes of Health (NIH), and were approved by the NINDS ACUC at NIH.

## Author Contributions

JL and WL designed the experiments and wrote the manuscript. JL, YL, and QL cloned and characterized sgRNA constructs and other plasmids. JL performed the biochemical and immunocytochemical experiments. JL, WH, and KW performed the electrophysiological assays. All authors read and commented on the manuscript.

## Conflict of Interest Statement

The authors declare that the research was conducted in the absence of any commercial or financial relationships that could be construed as a potential conflict of interest.

## Footnotes

1 http://crispr.mit.edu; http://www.e-crisp.org
